# Quantification and visualization of cellular uptake of TiO_2_ and Ag nanoparticles: comparison of different ICP-MS techniques

**DOI:** 10.1186/s12951-016-0203-z

**Published:** 2016-06-22

**Authors:** I-Lun Hsiao, Frank S. Bierkandt, Philipp Reichardt, Andreas Luch, Yuh-Jeen Huang, Norbert Jakubowski, Jutta Tentschert, Andrea Haase

**Affiliations:** Department of Chemical and Product Safety, German Federal Institute for Risk Assessment (BfR), Max-Dohrn-Strasse 8-10, 10589 Berlin, Germany; Department of Biomedical Engineering and Environmental Sciences, National Tsing Hua University, Hsinchu, Taiwan; Division of Inorganic Trace Analysis, German Federal Institute for Materials Research and Testing (BAM), Berlin, Germany

**Keywords:** Nanoparticles, Single particle ICP-MS, Laser ablation ICP-MS, Cellular internalization, Neurons

## Abstract

**Background:**

Safety assessment of nanoparticles (NPs) requires techniques that are suitable to quantify tissue and cellular uptake of NPs. The most commonly applied techniques for this purpose are based on inductively coupled plasma mass spectrometry (ICP-MS). Here we apply and compare three different ICP-MS methods to investigate the cellular uptake of TiO_2_ (diameter 7 or 20 nm, respectively) and Ag (diameter 50 or 75 nm, respectively) NPs into differentiated mouse neuroblastoma cells (Neuro-2a cells). Cells were incubated with different amounts of the NPs. Thereafter they were either directly analyzed by laser ablation ICP-MS (LA-ICP-MS) or were lysed and lysates were analyzed by ICP-MS and by single particle ICP-MS (SP-ICP-MS).

**Results:**

All techniques confirmed that smaller particles were taken up to a higher extent when values were converted in an NP number-based dose metric. In contrast to ICP-MS and LA-ICP-MS, this measure is already directly provided through SP-ICP-MS. Analysis of NP size distribution in cell lysates by SP-ICP-MS indicates the formation of NP agglomerates inside cells. LA-ICP-MS imaging shows that some of the 75 nm Ag NPs seemed to be adsorbed onto the cell membranes and were not penetrating into the cells, while most of the 50 nm Ag NPs were internalized. LA-ICP-MS confirms high cell-to-cell variability for NP uptake.

**Conclusions:**

Based on our data we propose to combine different ICP-MS techniques in order to reliably determine the average NP mass and number concentrations, NP sizes and size distribution patterns as well as cell-to-cell variations in NP uptake and intracellular localization.

**Electronic supplementary material:**

The online version of this article (doi:10.1186/s12951-016-0203-z) contains supplementary material, which is available to authorized users.

## Background

Due to their enhanced optical, electronical and antimicrobial properties, nanoparticles (NPs) are used in a variety of different consumer products like cosmetics, textiles or packaging and also in nanomedicine or electronics [1]. Titanium dioxide nanoparticles (TiO_2_ NPs) and silver nanoparticles (Ag NPs) belong to the group of highly commercialized NPs [2–5]. TiO_2_ NPs are frequently used as UV filters in cosmetics due to their absorption and scattering properties. However, TiO_2_ E171 is also used as a food additive, with a fraction of E171 being nanoscaled. Some types of TiO_2_ materials, in dependence on their crystal structure and chemical surface coating, have photocatalytic properties and are therefore used in sensors and self-cleaning surfaces [6, 7]. Ag NPs are often used because of their antimicrobial properties, e.g. in textiles but also for several medical applications such as wound dressings [8, 9]. Thus, human exposure to these kinds of NPs is increasing. Numerous in vivo and in vitro studies have evaluated the toxicity and toxic mechanisms of these NPs, which is summarized in several review articles [10, 11].

However, for safety assessment it is also important to analyze and quantify cellular uptake. Electron microscopy-based methods such as scanning electron microscopy (SEM) or transmission electron microscopy (TEM) can be used to localize NPs in intracellular ultrastructures with high spatial resolution. In combination with energy-dispersive X-ray spectroscopy (EDX) they also allow to identify the materials [12–14]. However, these techniques require extensive, time-consuming sample preparation and typically provide qualitative information [15]. Quantification might be also possible but requires time-consuming 3-D reconstruction and automated image analysis.

In cell biology, flow cytometry and/or fluorescent microscopy are often used for quantification of cellular uptake. In principle, both techniques are also applicable for fluorescent-labelled NPs [16, 17]. These techniques are easy to use and also allow for time resolved analysis. Possible drawbacks are dye leakage or possible surface modification due to dye-labelling, both of which may strongly influence the results and hence need to be considered beforehand [18]. An absolute quantification is not possible via these approaches.

Inductively coupled plasma mass spectrometry (ICP-MS) is a common technique for absolute quantification of the cellular uptake of metal or metal oxide NPs. It offers high sensitivity and selectivity for elemental analysis [19, 20]. Routinely it requires that cells are lysed before analysis. The absolute uptake per cell may be calculated after the measurement taking into account the cell number of the sample. However, this procedure reveals an average number only. In addition this approach will yield the total amount of the particular metal only. It will not allow to differentiate between single particles and agglomerated or ionic species. Also, it will not give information about the sizes of NPs. Despite these drawbacks ICP-MS is an established approach to quantify NP uptake and has been used in many studies. For instance it has been repeatedly found that the cellular uptake depends on the sizes of NPs [21, 22]. Mostly these studies present only calculated number concentrations and average amounts. Due to these limitations, little is known about real numbers of NPs per cell or about cell-to-cell heterogeneity. In the meantime, several modifications of ICP-MS became available that allow for single cell or single particle analysis [e.g. single cell ICP-MS (SC-ICP-MS) and single particle ICP-MS (SP-ICP-MS)] [14, 23, 24]. In SP-ICP-MS, highly diluted samples are measured. Since each signal in SP-ICP-MS corresponds to a single particle, the frequency of ICP-MS signals can be used to estimate the NP number concentration. On the other side, the intensity of signals is related to the amount of the chemical element and thus to the sizes of the respective NPs. When assuming spherical particle shapes, NP sizes and size distributions can be derived using SP-ICP-MS. For instance, the uptake of gold NPs in human umbilical vein-derived endothelial cells (HUVECs) was analyzed by SP-ICP-MS and a concentration-dependent increase in uptake was detected without agglomeration [14]. ICP-MS can be also coupled to laser ablation (LA-ICP-MS), which then allows the analysis of solid materials. This method has been applied to quantify gold, silver and aluminum oxide NPs at the single cell level [25–27]. The high spatial resolution of the LA-ICP-MS images also allows for sub-cellular localization of NPs or for detection of NP aggregation in cells [28].

**Fig. 1:**
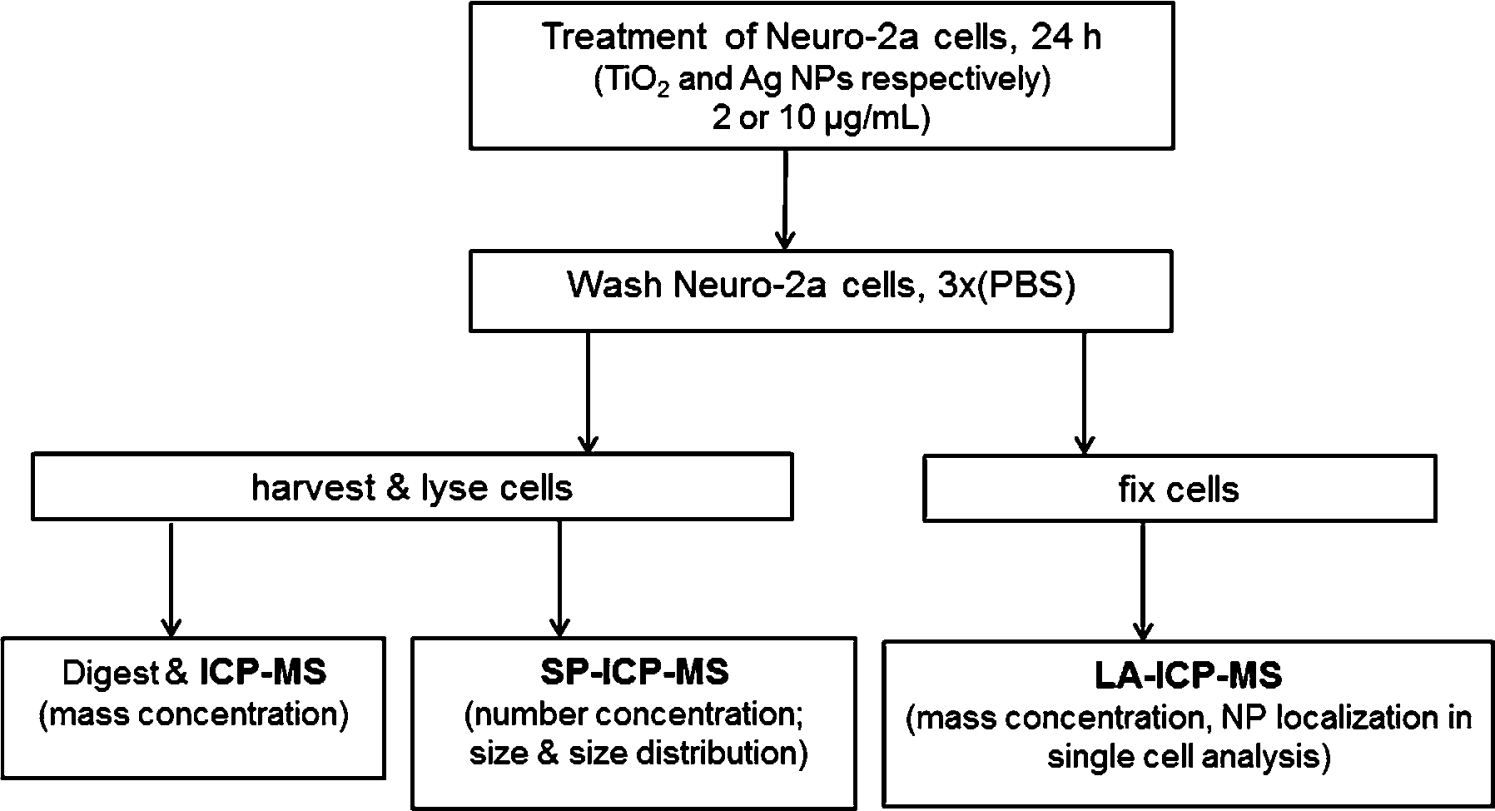
Work flow of this study

The work flow of our study is depicted in Fig. 1. The aim of the present study was to apply and to compare conventional ICP-MS, SP-ICP-MS, and LA-ICP-MS in the quantification of the cellular uptake of two types of TiO_2_ NPs (diameter of 7 and 20 nm, as determined by X-ray, respectively) and two types of Ag NPs (diameter of 50 and 70 nm, as determined by TEM, respectively). While the average NP mass concentration was determined by ICP-MS, average NP number concentration and size distribution was available from SP-ICP-MS. Cell-to-cell heterogeneity of NP uptake and intracellular distribution was analyzed by LA-ICP-MS. A differentiated Neuro-2a mouse neuroblastoma cell has been chosen because several studies indicated that NPs might be taken up into the central nervous system thereby exerting neurotoxic effects [29–32].

## Results and discussion

### Characterization of NPs

**Fig. 2:**
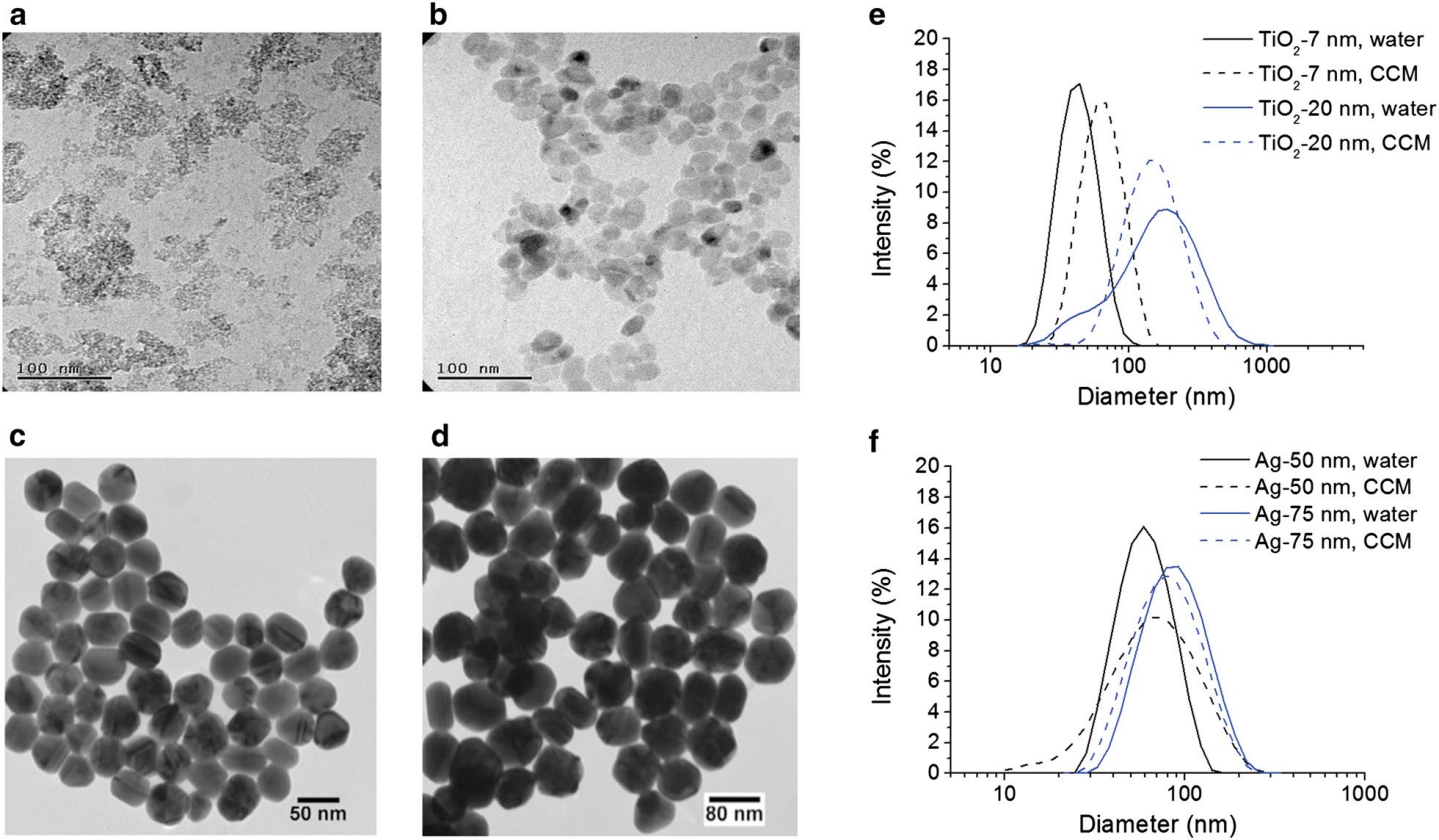
NP Characterization. TEM images of TiO_2_ 7 nm (**a**); TiO_2_ 20 nm (**b**); Ag 50 nm (**c**); Ag 75 nm (**d**). Images **c** and **d** were taken from NanoComposix with kind permission. Size distribution of TiO_2_ (**e**) and Ag NPs (**f**) in water and complete cell culture medium (CCM) as measured by DLS

We used two sizes of TiO_2_ NPs with diameters of 7 nm and 20 nm (as determined by X-ray), respectively. NP sizes were also analyzed by TEM (Fig. 2a, b). Their average hydrodynamic diameters in pure water was determined by dynamic light scattering (DLS). Size distribution in complete cell culture medium (CCM) containing serum was assessed by DLS (Fig. 2e) and nanoparticle tracking analysis (NTA) (Additional file 1: Figure S1). Data indicate some agglomeration for both types of TiO_2_ NPs but dispersion quality was still reasonably good. The alkali modification process for TiO_2_ NPs increases the density of surface hydroxyl groups and enhances their dispersion [33]. Furthermore, we used two sizes of Ag NPs with TEM diameters of 50 and 75 nm, respectively (Fig. 2c, d). Sizes determined by TEM was reported by the manufacturer. In addition, DLS was used to assess the average hydrodynamic diameter in pure water. Size distribution in CCM was investigated by DLS (Fig. 2f) and NTA (Additional file 1: Figure S1). Ag NPs were very well dispersed with only little sign of agglomeration visible using NTA. Material characterization is summarized in Table 1.

**Table 1:** Charaterization of the NPs used in this study

	X-ray (nm)	TEM (nm)	Dispersion in water	Dispersion in CCM		Dissolution in water (%)
			DLS (nm)	DLS (nm)	NTA (nm)	
TiO_2_ (7 nm), Anatase	7^a^	8 ± 2	48 ± 0.5	60 ± 0.5	43/78/143/283	NA
TiO_2_ (20 nm), Anatase	20^a^	35 ± 6	130 ± 4	128 ± 1.5	88/228	NA
Ag Cit (50 nm)		47 ± 5^b^	55 ± 0.6	57 ± 0.3	83 (Additional small peaks at 133 and 193)	1 ± 0.1 (10 μg/mL) 3.8 ± 0.9 (2 μg/mL)
Ag Cit (75 nm)		74 ± 8^b^	79 ± 0.5	71 ± 0.2	113 (Additional small peak at 188)	1 ± 0.03 (10 μg/mL) 3.1 ± 0.7 (2 μg/mL)

DLS data were presented as means including standard deviation. NTA data were reported as mode values. Dissolution was determined at 2 and 10 μg/mL. Dissolution of TiO_2_ NPs was not analyzed (NA)

^a^X-ray sizes of TiO_2_ NPs were reported by Ishihara Sanyo Kaisha, Ltd., Japan

^b^TEM values of Ag NPs were reported by NanoComposix, Prague, Czech Republic

### Assessment of NP-mediated cytotoxicity

Prior to the cellular uptake experiments, we carefully analyzed the cytotoxicity of the TiO_2_ and Ag NPs after 24 h of incubation (Additional file 1: Figure S2). The results show that both sizes of TiO_2_ NPs display no toxicity up to 25 µg/mL. Conversely, both sizes of Ag NPs showed a minimal toxicity at 25 µg/mL, with cell viabilities of 87 % for 50 nm and 90 % for 75 nm Ag NPs. According to these results, we used clearly non-cytotoxic doses in all subsequent experiments (i.e. 2 and 10 µg/mL of both Ag NPs and TiO_2_ NPs).

### Cellular uptake of NPs depends on particle size and concentration

We first performed calibrations using Ti and Ag ionic standards and In ions as internal standard. These resulted in linear correlations with squared correlation coefficients of (R^2^) >0.995 for both, Ti and Ag. The limit of detection (LOD) (as 3× STD+ background, n = 3) was determined at 0.05 pg Ag/cell and 0.31 pg TiO_2_/cell, respectively.

**Fig. 3:**
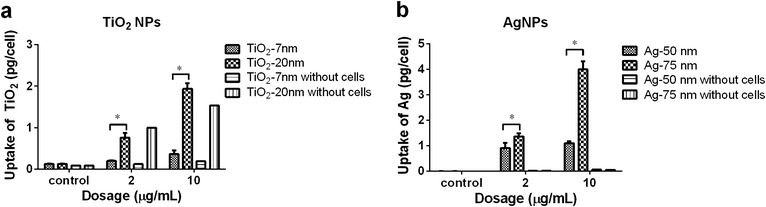
Cellular uptake of NPs in Neuro-2a cells as analyzed by ICP-MS. Depicted are data for TiO_2_ NPs (**a**) and Ag NPs (**b**). The ^49^Ti and ^107^Ag isotopes were selected for Ti and Ag detection, respectively. The internal standard was monitored using ^115^In. Analysis was conducted using a quadrupole ICP mass spectrometer, operated in standard mode. TiO_2_ was also detected in samples without cells, which indicates that NPs physically attach to cell culture plates and cannot be completely removed by subsequent washing with PBS. (*) indicates a significant difference between two treatment groups as determined by student’s t test (p < 0.05)

Using these calibrations, we quantified the cellular uptake by measuring the total amounts of Ti and Ag after digestion of Neuro-2a cells upon treatment with two concentrations, i.e. 2 and 10 μg/mL (Fig. 3a, b).

Higher mass amounts of TiO_2_ and Ag were taken up after incubation with larger particles compared to the smaller ones. Furthermore, uptake was increased at higher concentrations. For the TiO_2_ NPs this resulted in a 1.9 and 2.6-fold higher cellular Ti amount for the 7 and 20 nm NPs at 10 µg/mL when compared to 2 µg/mL, respectively (Fig. 3a). In the case of Ag NPs only the 75 nm (TEM) showed a clear increase in uptake after treatment with the higher incubation concentration (2.9-fold) (Fig. 3b). To rule out the possibility that the detected signals were due to NPs that physically attached to the surface of the cell cultures plates, we performed control treatments without any cells, which were analyzed accordingly (Fig. 3a, b). After identical sample preparation in the controls almost no Ag could be detected (Fig. 3b). However, some Ti was detected, which is likely to represent the fraction of NPs that unspecifically attached to the cell culture plates and which could not be completely removed by washing (Fig. 3a). In the case of 10 µg/mL of 7 nm TiO_2_ NPs (X-ray), the numbers were 109 ng total Ti in the presence of cells (equals 0.36 pg/cell) versus 46 ng in the absence of cells (equals 0.19 pg/cell when assuming the same cell number as in the treatment group). Similarly, for 10 µg/mL of 20 nm TiO_2_ NPs (X-ray) 559 ng of Ti was detected when cells were present (equals 1.93 pg/cell), whereas 443 ng were detected in controls (equals 1.53 pg/cell).

**Fig. 4:**
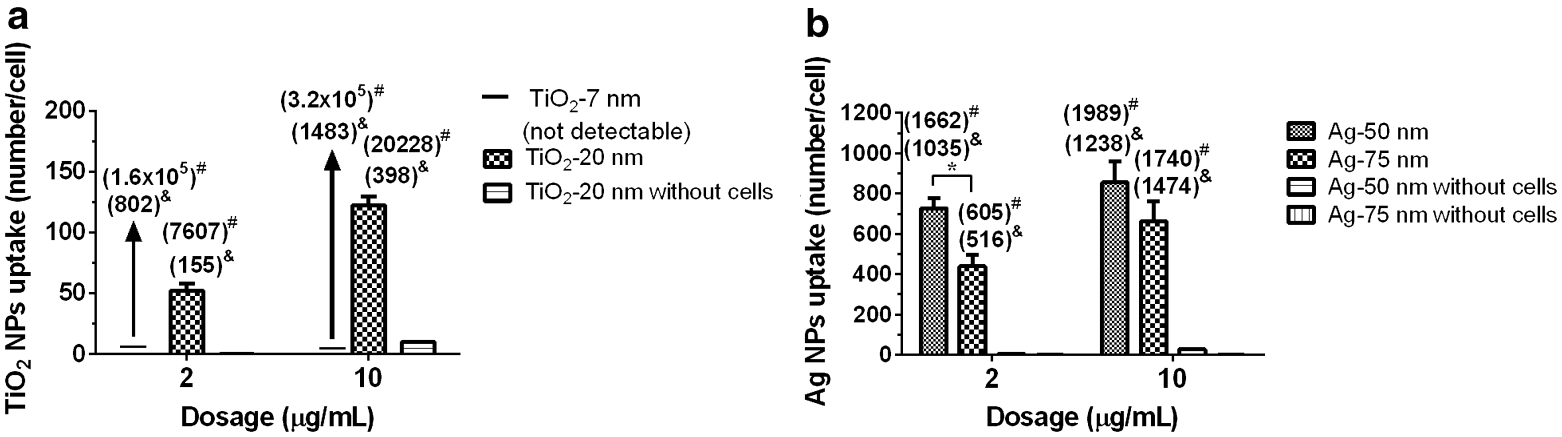
Number-based cellular uptake of NPs in Neuro-2a cells as analyzed by SP-ICP-MS. Depicted are data for TiO_2_ NPs (**a**) and Ag NPs (**b**). The ^48^Ti and ^107^Ag isotopes were selected for Ti and Ag detection, respectively. Analysis was conducted using a quadrupole ICP mass spectrometer, operated in spike mode (1 min per run, dwell time of 3 ms per reading). The small TiO_2_ NPs cannot be detected by SP-ICP-MS because their size was below the limit of detection size (LOD_size_) of TiO_2_ (69 nm). ^#^Calculated number of NPs per cell using ICP-MS (mass) data. ^&^Calculated number of NPs per cell using ICP-MS (mass) data based on DLS size. (*) indicates a significant difference between two treatment groups as determined by student’s t-test (p < 0.05)

Furthermore, we used SP-ICP-MS to quantify the NP numbers in Neuro-2a cells directly, which was, according to our knowledge, investigated here for the first time. First, we performed a calibration experiment by using NP suspensions. The LOD_size_ and LOD_numberNP_ of Ag NPs was 22 nm and 2.26 × 10^5^ particles, respectively. The LOD_size_ and LOD_numberNP_ of TiO_2_ NPs was 69 nm and 1.92 × 10^5^ particles, respectively. Therefore, the 7 nm TiO_2_ NPs with a secondary size of 48 nm (DLS) could not be detected. However, the 20 nm TiO_2_ NPs (X-ray) as well as both types of Ag NPs could be quantified via SP-ICP-MS. The 20 nm TiO_2_ NPs (X-ray) showed a concentration-dependent uptake into Neuro-2a cells as indicated by the SP-ICP-MS measurements (Fig. 4a). In addition to the concentration dependency, for Ag NPs we were able to confirm a size dependency as well, as the number of NPs per cell increased with decreasing particle size (Fig. 4b). Therefore, the smaller Ag particles can penetrate into cells more efficiently.

Assuming ideal spheres and no dissolution for both types of NPs, the NP numbers per cell can also be calculated using the results from ICP-MS. These results confirmed that the treatment with the smaller TiO_2_ and Ag NPs resulted in higher particle numbers per cell (Fig. 4a, b). However, assuming the TEM size as single particle diameter, the particle numbers calculated based on ICP-MS data were considerably higher when compared to the particle numbers directly measured by SP-ICP-MS using the same lysates. Between calculated and measured NP numbers the differences were 100-fold for TiO_2_ and twofold for Ag NPs (Fig. 4a, b). By contrast, assuming DLS size as single particle diameter, the differences between calculated and measured NP numbers decreased to about three- to fourfold for TiO_2_ and 1.1- to 1.4-fold for Ag NPs. This result indicated that NP sizes determined by DLS were more suitable for the transformation of NP amounts into NP numbers, when compared to NP sizes determined via TEM or X-ray. Indeed, the differences between estimated and measured NP numbers may be explained, at least partially, by NP agglomeration in cells, which was found higher in the case of TiO_2_ NPs. This aspect will be discussed in the following section in more detail.

### LA-ICP-MS also confirms the size-dependent cellular uptake of NPs

For NP quantification by LA-ICP-MS we first analyzed the calibration curve, which was based on adding NPs to a cell lysate of untreated cells (Additional file 1: Figure S3a). A laser spot size of 250 μm (line distance of 240 μm) and a scan speed of 200 μm/s were considered to be optimal LA parameters. For silver, the calibration range comprised five Ag-containing spots and one control spot without Ag. The latter was pipetted at around X = 2.5 mm but could not be distinguished from the background of the glass slide. A background correction for each drop was performed. Since the upper and lower area showed a different background signal, both replicates were corrected separately. A good correlation (R^2^ = 0.995) between the amount of silver and the integrated signal intensity was observed and a limit of detection of 78.5 pg was calculated (Additional file 1: Figure S3b).

LA-ICP-MS in general is able to provide high spatial localization information (1 μm pixel size in *x*-direction and 6 μm in *y*-direction) as reported before by Drescher and co-workers analyzing Au and Ag NP aggregates in the mouse fibroblast 3T3 cell line [25]. A laser spot size of 4 μm (line distance of 6 μm) and a scan speed of 5 μm/s was used. As Neuro-2a cells were smaller compared to 3T3 cells this may result in a much lower number of pixels to represent the NPs in a single cell but does not affect the integrated sensitivity under the same conditions. Therefore, for Neuro-2a cells a reasonable sub-cellular localization requires further optimization of the ablation parameters. However, by decreasing the line distance to 5 μm (1 μm pixel size in *x*-direction and 5 μm in *y*-direction in spatial resolution) we could show that no NPs were localized in the cell neurites. Neurites propagate the signals between neural cells and are usually not responsible for uptake. However, we may not entirely exclude that NP concentrations in neurites were below the detection limits of LA-ICP-MS.

**Fig. 5:**
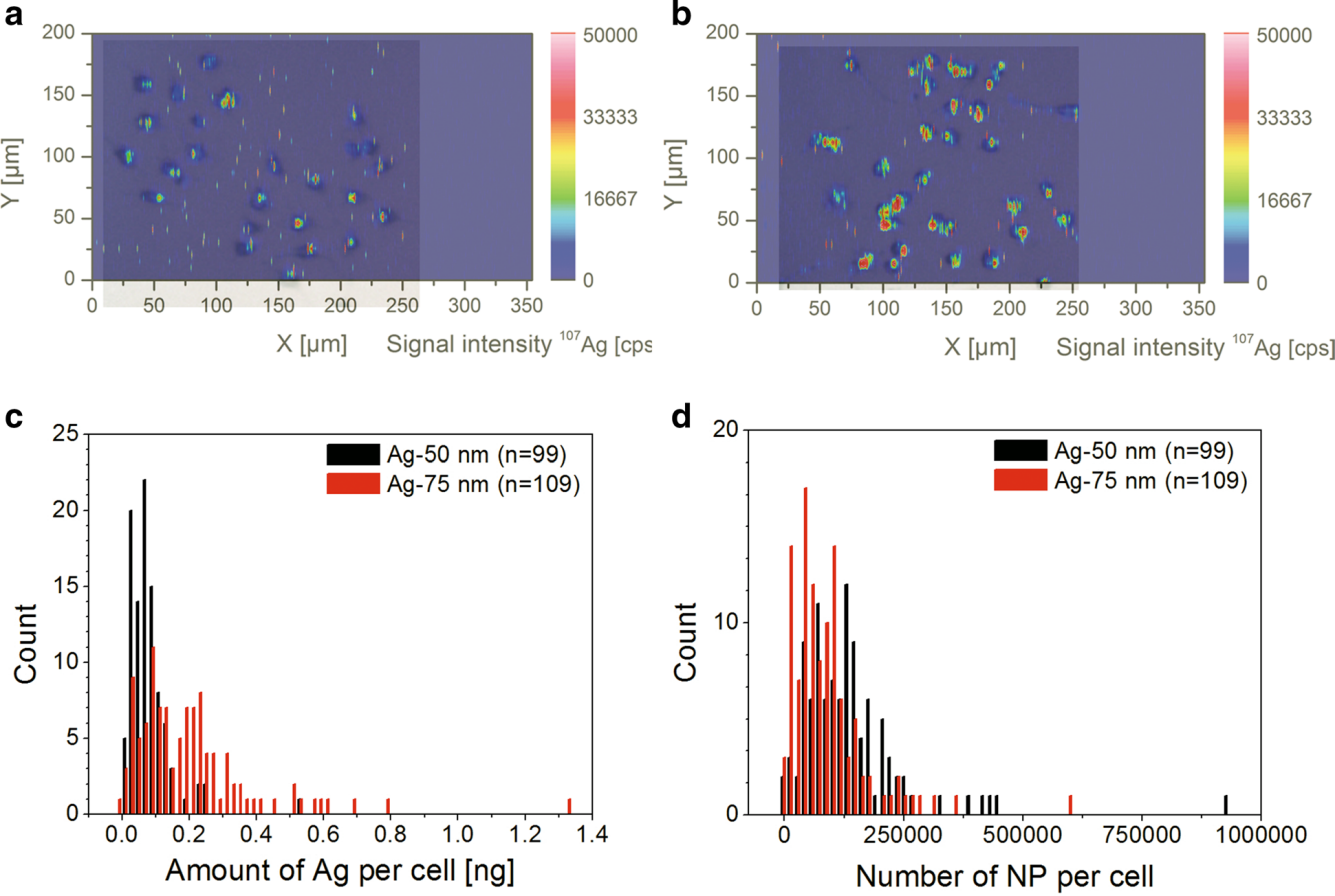
Cellular uptake of Ag NPs at single cell level as analyzed by LA-ICP-MS. Depicted are overlapping contour plots of ^107^Ag with cell morphology of Neuro-2a cells after incubation with 2 μg/mL 50 nm (**a**) and 75 nm (**b**) Ag NPs. Histogram for the amount of Ag determined in single cells upon incubation with Ag NPs (bin width: 0.02 ng) (**c**). Histogram of the numbers of Ag NPs per cell (bin width: 15,000 NPs) (**d**). Histograms (**c**) and (**d**) were calculated from three ablated areas on the slide

The contour plots for the ^107^Ag signal intensities of the dried cells nicely corresponded with bright field images of Neuro-2a cells before ablation (compare Fig. 5a, b). We found that cells after incubation with 75 nm Ag NPs (TEM) often revealed more ^107^Ag spots with a higher intensity compared to cells treated with 50 nm Ag NPs (TEM). Furthermore, 75 nm Ag NPs (TEM) could frequently be detected outside the cells while 50 nm Ag NPs (TEM) were mainly detected within the cell areas only.

Signals from 99 and 109 cells from several ablated areas were integrated after treatment with 50 and 75 nm Ag NPs (both TEM sizes), respectively. Using the calibration curve, the calculated amounts of silver per cell after a 2 µg/mL incubation ranged from 0.006 to 0.528 ng for 50 nm Ag NPs (0.081 ± 0.067 ng in average), and from 0.001 to 1.332 ng for 75 nm Ag NPs (0.205 ± 0.188 ng in average), respectively (Fig. 5c). This again confirmed a higher NP mass per cell for the bigger 75 nm particles and at the same time demonstrates a high cell-to-cell variability, which seems increasing at higher treatment concentrations.

Assuming ideal spheres, DLS size as particle diameter, consisting purely of silver for both nanoparticles types, the total silver amounts per cell were converted into NP numbers (Fig. 5d). Again, a higher NP number per cell was found after incubation of the cells with the smaller 50 nm Ag NPs (TEM) (see Table 2).

**Table 2:** Average and median values of NP uptake in single Neuro-2a cells

Sample/unit	Median	Average ± SD
50 nm Ag (pg per cell)	72	81 ± 67
50 nm Ag (NPs per cell)	126,085	142,394 ± 117,729
75 nm Ag (pg per cell)	164	205 ± 188
75 nm Ag (NPs per cell)	73,684	92,152 ± 84,502
7 nm TiO_2_ (integrated signal intensity cps per cell)	107,242	136,234 ± 103,648
7 nm TiO_2_ (relative NPs per cell)	4.8 × 10^11^	6.0 × 10^11^ ± 4.6 × 10^11^
20 nm TiO_2_ (integrated signal intensity cps per cell)	281,341	310,732 ± 203,387
20 nm TiO_2_ (relative NPs per cell)	6.3 × 10^10^	6.9 × 10^10^ ± 4.6 × 10^10^

The cellular uptake was analyzed by LA-ICP-MS. Exposure concentration: 2 µg/mL

Using the same strategy as for the Ag NPs, we could not achieve an according calibration for the TiO_2_ NPs though. The ^48^Ti signal spots for the pipetted calibration spots could not be detected and integrated clearly. Instead the spot areas showed a depleted signal intensity compared to a relatively high and irregular background (data not shown). Higher spots within these areas might indicate some flow mechanism for the TiO_2_ NPs similar to the coffee stain effect [34, 35]. As our calibration was not successful, we can only provide ^48^Ti signal intensities in this study but no quantification. For further studies one may consider to use aqueous Ti standards instead of TiO_2_ NPs.

**Fig. 6:**
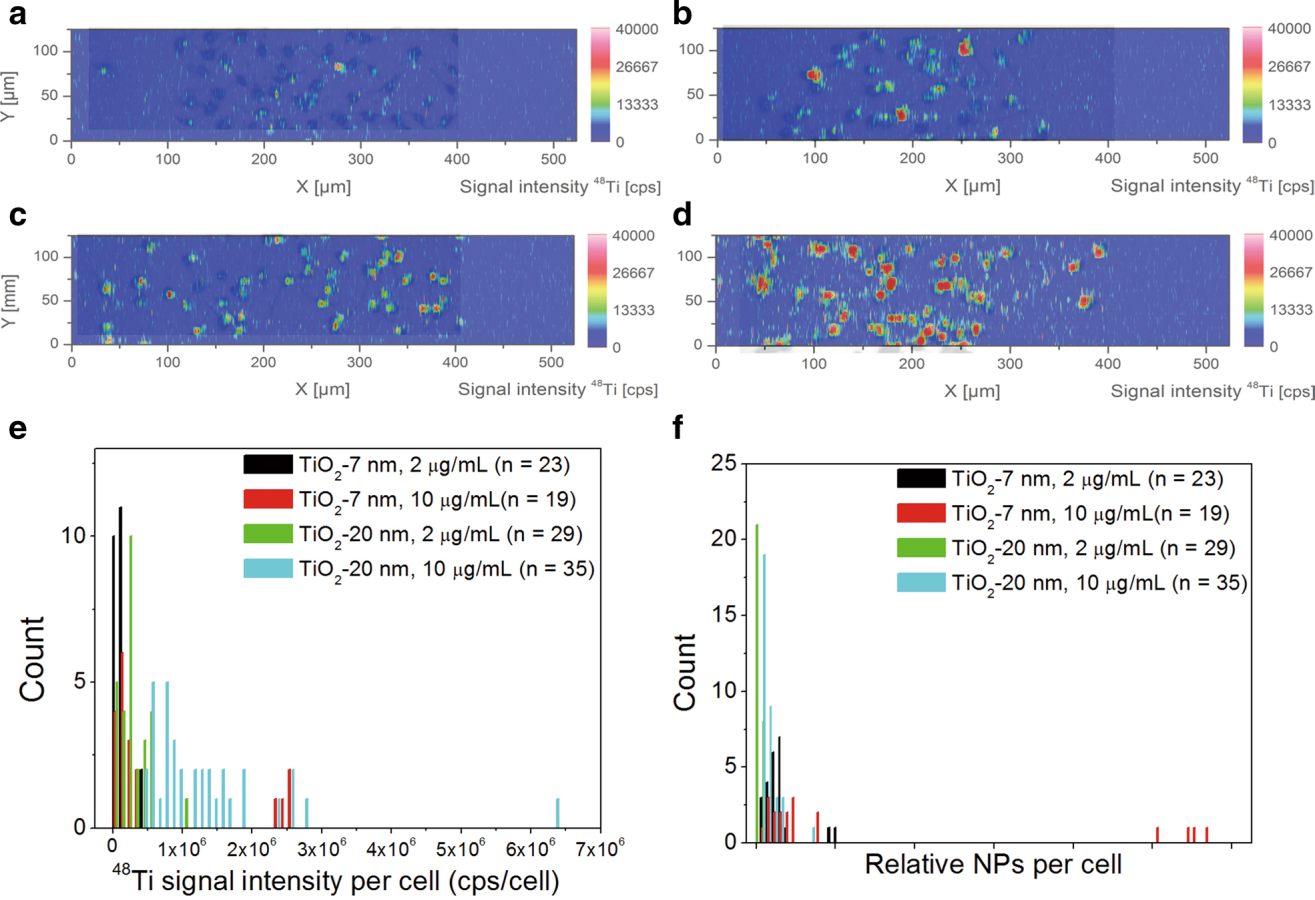
Cellular uptake of TiO_2_ NPs at single cell level as analyzed by LA-ICP-MS. Depicted are overlapping contour plots of ^48^Ti with cell morphology of Neuro-2a cells after incubation with TiO_2_ NPs: 7 nm, 2 μg/mL (**a**); 7 nm, 10 μg/mL (**b**); 20 nm, 2 μg/mL (**c**); and 20 nm, 10 μg/mL (**d**). Histogram for the cps of TiO_2_ determined in single cells upon incubation with TiO_2_ NPs (bin width: 0.02 ng) (**e**). Histogram of the relative numbers of TiO_2_ NPs per cell (bin width: 2000 NPs) (**f**)

Figure 6a–d show exemplarily the overlaps of the contour plots for the ^48^Ti signals with the corresponding bright field images of the dried Neuro-2a cells. In agreement with prior results, more ^48^Ti signal spots in each cell were observed after higher concentrated incubations (10 µg/mL) compared to lower concentrations (2 µg/mL). Similarly, more ^48^Ti signal spots could be detected for larger TiO_2_ NPs (20 nm, X-ray). Part of the titanium signal detected using conventional ICP-MS may stem from particles that unspecifically attached to the surface of the cell culture plate, which is causing an overestimation of cellular uptake. Using LA-ICP-MS allows to selectively integrate only signals within the cells thereby correcting any background and eliminating this error.

Integration of the dried cells showed that after incubation with a higher NP concentration or larger NPs, a higher signal intensity of ^48^Ti can in average be found per cell which equals a higher amount (Fig. 6e). The 10 µg/mL incubation resulted for both NP sizes in an approximately 4.5-fold increased signal intensity. The larger 20 nm TiO_2_ NPs led to twice the signal intensity, independent of the used NP concentration (Fig. 6e).

**Fig. 7:**
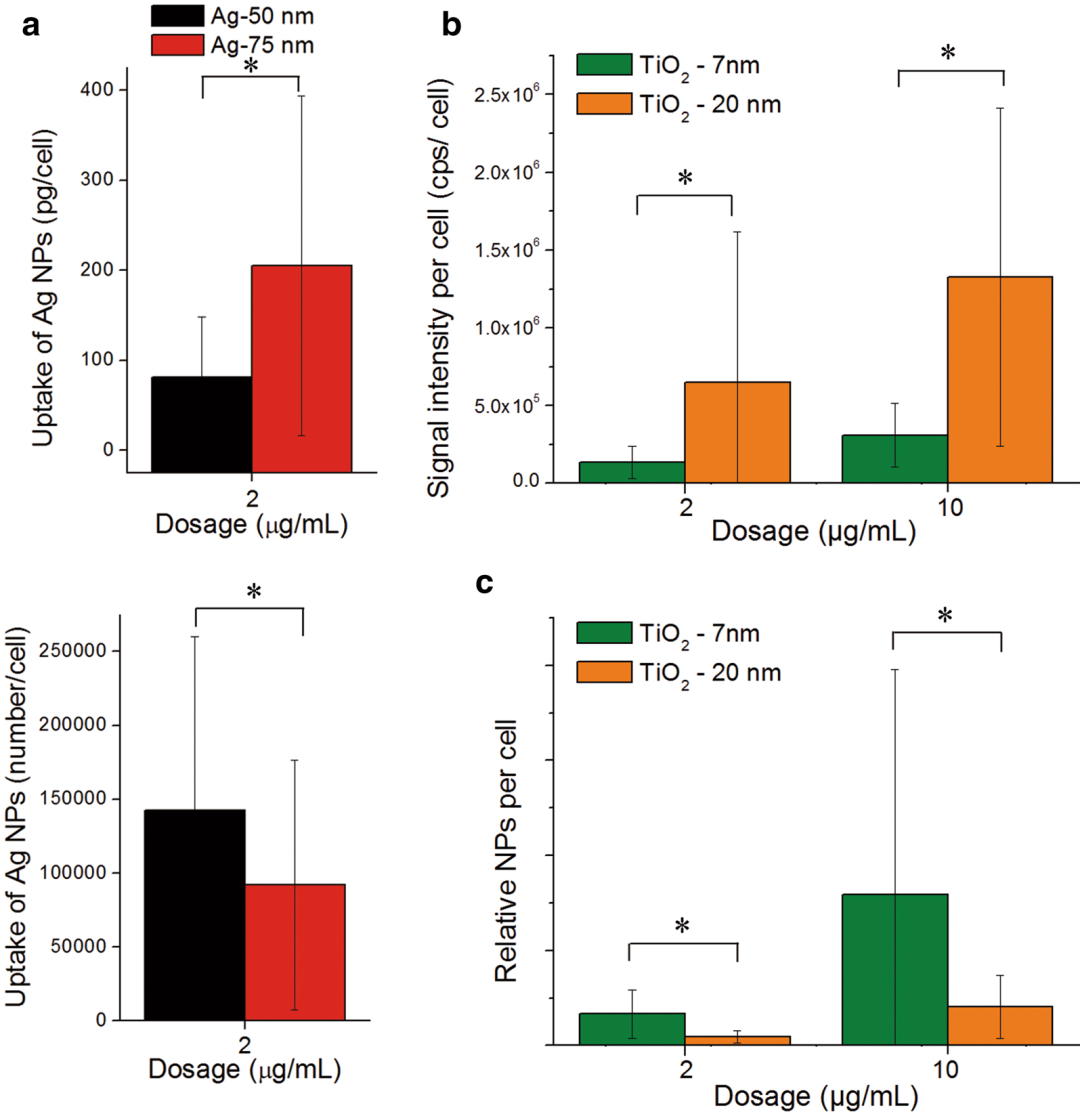
Quantification of cellular uptake of Ag and TiO_2_ NPs in Neuro-2a cells as analyzed by LA-ICP-MS. Average cell uptake based on particle mass (pg/cell) and particle number (Ag NPs/cell) upon exposure to 2 μg/mL Ag NPs (**a**). Average signal intensity of ^48^Ti per cell upon exposure of cells to 2 or 10 μg/mL TiO_2_ NPs (**b**), and corresponding average relative TiO_2_ NP numbers per cell (**c**). (*) indicates a significant difference between two treatment groups as determined by student’s t-test s (p < 0.05)

Despite the fact that calibration failed for the TiO_2_ NPs, we could use our data to obtain the relative numbers of particles per cell. Utilizing the DLS size ratio between both TiO_2_ NP types, the integrated signals of ^48^Ti per cell were converted to relative NP numbers—only missing the calibration factor (cps/ng)—and could be compared. Figure 6f shows the distribution of the relative numbers of particles per cell for each exposure condition. On average, the relative number of the smaller TiO_2_ NPs per cell was found to be four times the number of the larger TiO_2_ NPs (Table 2; Fig. 7). This again confirms that smaller particles are more easily incorporated into cells. At the same time a big variance in the numbers of NPs per cell was seen especially after incubation with the higher concentration (10 µg/mL). This indicates a cell-to-cell diversity, which may be even increased at higher treatment concentrations.

Using LA-ICP-MS we could detect even the smaller TiO_2_ NPs with a diameter of 7 nm (X-ray) above background levels, which was not the case in the corresponding SP-ICP-MS experiments.

### NP agglomerates in cells can be detected using SP-ICP-MS

In addition to particle numbers, SP-ICP-MS can also be applied to estimate NP size distributions. A cell-free 20 nm TiO_2_ NP suspension in ultrapure water revealed an average diameter of 115 nm and a size range of 76–260 nm (Additional file 1: Figure S4a, b). This size value was close to the size value determined by DLS (130 nm). However, in cell lysates the average NP size value increased to 134 nm with size ranging from 76 to 300 nm, especially for the 10 µg/mL incubation (Additional file 1: Figure S5a, b). Cell-free 50 and 75 nm Ag NP suspensions (both TEM sizes) in pure water showed 46 and 72 nm in average diameter, respectively (Additional file 1: Figure S4c–f). Again, in cell lysates, average sizes of both Ag NPs also increased to 55 and 84 nm at 10 µg/mL because more particles could be detected in the range of 150–200 nm (Additional file 1: Figure S5c-f). This indicates that NP agglomerates are being formed in Neuro-2a cells after particle uptake. Such agglomerates of NPs, ranging from submicron to micron size, can frequently be found in cells by different electron microscope techniques like TEM [36, 37]. These data correspond well with lower numbers of NPs measured by SP-ICP-MS as theoretically calculated based on conventional ICP-MS data. One reason for this discrepancy is likely the agglomeration of both NP types. Agglomeration is more pronounced for TiO_2_, which nicely conforms with the higher difference between calculated and measured NP numbers for TiO_2_ NPs. In addition, Ag NPs could dissolve into silver ions. We observed a higher background noise for cell samples treated with Ag NPs compared to control cells in pure water (data not shown), which may be due to Ag NP dissolution. Finally, for TiO_2_ the LOD_size_ hinders the detection of small TiO_2_ particles by SP-ICP-MS and may thereby also affect the results.

Studies applying conventional ICP-MS have already found a size-dependent uptake of TiO_2_ and Ag NPs in mammalian cells [38, 39]. However, in these studies frequently smaller NPs were reported to show a higher mass concentration in cells, which is in contrast to the results in our study. Zhu et al. analyzed uptake of silica NP by atomic absorption spectrometry (AAS) in HeLa cells, and found a higher uptake efficiency for smaller SiO_2_ NPs in both mass and estimated number concentrations [40]. Furthermore, some studies reported no size-dependency of the uptake, as shown for citrate-coated Ag NPs and human lung cells analyzed by AAS [13]. These studies rely on indirect, average results. Our study clearly showed a size-dependent uptake for both Ag and TiO_2_ NPs in Neuro-2a cells. However, we find a higher mass concentration for the larger particles but a higher particle number for the smaller NPs, consistently across different techniques. The different results between all these studies may also be caused by different uptake mechanisms of NPs in different cell types. Furthermore, differences may arise from different surface modifications. Therefore a general conclusion might not be possible at the moment until the underlying mechanisms of cellular uptake are fully understood. The main features of each technique applied in our study is summarized in Table 3.

**Table 3:** Specific features of the different ICP-MS techniques applied in the present study

Technique	Parameter	Size	Localization	Limitations	Limit of detection (LOD)
ICP-MS	Mass	No	No	Error from NPs attached to culture plates	Ag: 0.05 pg/cell TiO_2_: 0.31 pg/cell
SP-ICP-MS	Number	Yes	No	Spherical NPs only, high LOD_size_ for TiO_2_	Ag: 2.26 × 10^5^ particles (22 nm)^a^, TiO_2_: 1.92 × 10^5^ particles (69 nm)^a^
LA-ICP-MS	Mass in single cell (relative measure)	No	Yes	Localization for small cells difficult	Ag: 78.5 pg per pippetted spot of 0.5 µl TiO_2_: not available in this study

^a^Diameter of NPs was calculated from LOD

## Conclusions

In the present study, ICP-MS, SP-ICP-MS, and LA-ICP-MS were used to quantify the uptake of TiO_2_ NPs or Ag NPs in neuroblastoma cells. While cellular uptake was clearly NP size-dependent, we consistently found a higher mass concentration in the case of the larger NPs but a higher particle number for the smaller NPs. ICP-MS provides average mass quantification data, which can be converted into NP numbers. The results may, however, be confounded by NP agglomeration inside cells, resulting in an overestimation of NP numbers. Furthermore, NPs may unspecifically attach to the surface of the cell culture dishes and withstand the regular washing steps prior to analysis. This again would lead to an overestimation of the NP amounts in conventional ICP-MS. In particular this was observed for the cellular uptake of TiO_2_ NPs in our study. SP-ICP-MS directly measures NP numbers. Furthermore, using SP-ICP-MS it is possible to estimate size distributions of spherical particles directly in cell lysates. This allows to analyze possible NP agglomeration inside cells. However, smaller TiO_2_ particles cannot be measured due to the high LOD_size_ value. By LA-ICP-MS it becomes possible to directly quantify NP mass concentrations at single cell level as done here for Ag NPs. On the other side, we observed difficulties in the calibration of TiO_2_ NPs, so that we only could derive relative signal intensities. Notwithstanding applying LA-ICP-MS it is possible to obtain information on the sub-cellular localization of particles. As each technique offers specific advantages we propose to combine them in order to successfully elucidate different aspects of NP’s cellular uptake.

## Methods

### Materials

Ultrapure water (18.2 MΩ/cm) was generated by a Milli-Q Plus system from Merck Millipore (Darmstadt, Germany). The Ag elemental standard as well as nitric acid (p. A., 65 %) were purchased from Merck (Darmstadt, Germany). The Ti and In elemental standard were purchased from Sigma-Aldrich (TraceCERT^®^, Steinheim, Germany). Hydrogen peroxide (H_2_O_2_) solution (p.A., 30 %), sodium hydroxide (NaOH), Triton X-100 solution, forskolin, 3-isobutyl-1-methylxanthine (IBMX), and paraformaldehyde were purchased from Sigma-Aldrich (Steinheim, Germany). Hydrochloric acid (p.A., 37 %) was bought from Applichem (Darmstadt, Germany). Hydrofluoric acid (p.A., 5 %) was bought from Carl Roth (Karlsruhe, Germany). SuperFrost^®^Plus Slides were used as glass slides for cell culture LA-ICP-MS experiments (Thermo Fisher Scientific GmbH, Dreieich, Germany).

#### Nanoparticles

Commercially available 50 and 75 nm Ag nanospheres, carrying a citrate modification, were utilized (NanoComposix, Prague, Czech Republic). Alkali-modified TiO_2_ NPs were synthesized as described in a previous study [33]. Briefly, 1 g TiO_2_ commercial nanopowder (ST-01, 7 nm or ST-21, 20 nm, 100 % anatase, Ishihara Corporation, Japan) was dispersed in a 100 mM H_2_O_2_ and 8 M NaOH mixed aqueous solution. Then, the suspension was heated to 50 °C and kept for 24 h. After centrifugation at 8000 rpm for 20 min, the particles were washed twice with de-ionized water and re-dispersed in a 1.6 M HNO_3_ solution. The suspension was stirred for 3 h and then centrifuged at 13,500 rpm for 20 min. After three times wash with de-ionized water, the TiO_2_ NPs were dried at 60 °C and re-dispersed in water for subsequent experiments. Final concentrations of the suspension were determined by inductively coupled plasma optical emission spectrometry (ICP-OES) (Agilent 725, Santa Clara, USA). Stock solutions of Ag NPs and TiO_2_ NPs were first ultrasonicated for 5 min and then diluted to 100 μg/mL in the indicated medium. All NP suspensions were freshly prepared before each experiment.

#### Characterization of nanoparticles

The particle size and morphology of the alkali-modified TiO_2_ NPs in suspension were analyzed using a JEM2100 transmission electron microscope at an acceleration voltage of 200 kV (TEM) (JEOL, Japan). The hydrodynamic size of the Ag NPs and TiO_2_ NPs in stock solution and cell culture medium (CCM) were monitored using a Zetasizer Nano ZS apparatus (Malvern Instruments GmbH, Herrenberg, Germany). Particle solutions were diluted for the DLS measurement to a final concentration of 100 μg/mL for TiO_2_ NPs and 10 μg/mL for Ag NPs. For nanoparticle tracking analysis (NTA) we used a NanoSight LM20 system (Malvern, Germany) equipped with a green laser (532 nm) at RT (22 °C). Sizes were calculated from videos (60 s, 30 frames per s) by NTA3.1 software. Fetal calf serum (FCS) contained numerous agglomerates, which disturbed analysis. Therefore, NP dispersions were prepared in CCM with final concentrations of 10 μg/ml for nanosilver and 100 μg/ml for TiO2 and then diluted 1:40 into protein-free MEM such that the serum-agglomerates were not detectable any more. Proteincorona of NPs were considered as rather stable, not likely to disintegrate during the time period of NTA measurements. Nanosilver dissolution in water was determined using ICP-MS as described below. Two different concentrations were tested, i.e. 2 and 10 μg/ml. Suspensions were incubated for 24 h at 37 °C and were then centrifuged at a table top centrifuge at 16,000×*g* for 30 min to remove NPs. Supernatants were filtered through Amicons filters (cut off 30 kDa) and then processed as described below for ICP-MS analysis.

#### Cell culture

Mouse neuroblastoma (Neuro-2a) cells (Cell Lines Service GmbH, Eppelheim, Germany) were cultured in MEM medium (Gibco, Darmstadt, Germany) supplemented with 10 % fetal calf serum (FCS) (Pan Biotech, Aidenbach, Germany), 2 mM l-glutamine, 0.1 mM non-essential amino acids, and 1.0 mM sodium pyruvate (Gibco, Darmstadt, Germany). Cells were cultivated at 37 °C, 5 % CO_2_ and 95 % relative humidity. Twenty four hours after seeding, cells were differentiated using 30 μM forskolin and 200 μM 3-isobutyl-1-methylxanthine (IBMX) (both obtained from Sigma-Aldrich, Steinheim, Germany) in MEM/1 % FCS medium for 2 days into neuronal-like cells.

#### Cytotoxicity

WST-1 cell viability assay was used to evaluate the toxicity of TiO_2_ NPs and Ag NPs according to manufacturer’s instructions (Roche Diagnostics, Mannheim, Germany). Neurite-bearing cells (1.8 × 10^4^ cells/cm^2^) were treated with 5, 10 and 25 μg/mL TiO_2_ NPs or Ag NPs, respectively, in 96-well plates for 24 h. Interfering NPs were removed in a table top centrifuge by centrifugation with maximum speed prior to spectrophotometric read-out (TECAN, Crailsheim, Germany) at 450 nm.

#### Cell incubation and sample preparation

For analysis by ICP-MS and SP-ICP-MS, cells were seeded and differentiated in 12-well plates (1.8 × 10^4^ cells/cm^2^). They were exposed to 2 or 10 μg/mL NPs in MEM/5 % FCS medium for 24 h. It should be noted, that in vitro test concentrations in the range from 1 to 10 µg/cm^2^ correlate very well to test concentrations usually used in in vivo inhalation studies and in particular they correlate well to the overload dose, i.e. the dose where toxic effects become detectable. Therefore, in vitro test concentrations in the range from 1 to 10 µg/cm^2^ are useful for comparing the data later on to results obtained in in vivo experiments.

Before analysis cells were washed three times with DPBS (Dulbecco’s Phosphate Buffered Saline) before being trypsinized and harvested by centrifugation (250×*g*, 5 min). In each case, 10 μL were used to count cells. Cells were lysed in 1 mL 0.1 % Triton X-100 (Sigma, Taufkirchen, Germany). Half of each lysate was analyzed by SP-ICP-MS. The other half was digested for ICP-MS. The cell lysates and digested solutions were stored at 4 °C before analysis. Control samples without any cells were prepared using the same protocol.

For LA-ICP-MS, the cells (1 × 10^4^ cells/cm^2^) were grown on polymer coated glass slides and incubated with NPs (2 µg/mL for Ag NPs or 2 and 10 µg/mL for TiO_2_ NPs) for 24 h. Cells were washed three times with DPBS and fixed with 4 % paraformaldehyde in DPBS. Therafter they were dehydrated in a series of ethanol (15, 30, 50, 70, 90 and 99.5 %). The slides were stored at 4 °C before analysis. Control cells without NPs were prepared similarly.

#### ICP-MS analysis of digested cells

For Ag NPs, 10 mL 65 % HNO_3_ was used for digesting cell lysates. For TiO_2_ NPs, 2 mL 65 % HNO_3_ and 4 mL 5 % HF was used. Digestion was performed in open vials at 60 °C using a heating block for 30 h. Total amounts of titanium and silver in the cells were determined by ICP-MS using a quadrupole ICP mass spectrometer (XSeries 2, Thermo Fisher Scientific GmbH, Dreieich, Germany). The ICP-MS parameters were optimized and are given in Additional file 1: Table S1. For the Ti and Ag detection, the ^49^Ti and ^107^Ag isotopes were selected. As an internal standard ^115^In was used. Digested samples were filled up with ultrapure water to 50 mL with 10 ppb of ^115^In as internal standard before analysis. Calibrations were performed using ionic Ti and Ag standards in 6.5 % HNO_3_ solution ranging from 0 to 20 μg/L for both elements. All experiments were conducted in three independent replicates for each treatment and control group. For the calculation of the number of NPs per cell, a perfect sphere was assumed for the particles and either TEM or DLS particle diameters were used.

#### SP-ICP-MS analysis of cell lysates

SP-ICP-MS was used to analyze number and size of NPs in the cell lysates (XSeries 2, Thermo Fisher Scientific GmbH, Dreieich, Germany). The SP-ICP-MS parameters were optimized and are given in Additional file 1: Table S1. Before analysis, the lysates were homogenized by vortexing and then diluted in ultrapure water to 50–100 ng/L Ag or Ti based on the ICP-MS measurements of digested cells, which were performed beforehand. For quality control of the nebulizer transport efficiency a freshly prepared 60 nm AuNP standard (NIST-RM 8013, Gaithersburg, MD, USA) was used at a concentration of 100 ng/L in ultrapure water, which yielded an average size of 61 nm (Additional file 1: Figure S6). The nebulizer transport efficiency ranged from 0.039 to 0.045.

For all measurements the instrument was operated in spike mode separately detecting ^197^Au, ^107^Ag and ^48^Ti isotopes one at a time. A constant sample flow rate of 0.34 mL/min was utilized. The duration time for each run was set at 1 min with a dwell time of 3 ms per reading. To determine the detector sensitivity in SP-ICP-MS for both elements, one point of each calibration series established by ICP-MS for digested cells (10 µg/L for Ag and Ti ion standard) was measured in spike mode. The limits of detection regarding the NP size (LOD_size_) of the TiO_2_ NPs and Ag NPs were calculated as described as follows with a 3σ threshold to identify the particle signal from the background noise [41]:

$$ {\text{LOD}}_{\text{size}} = \left( {\frac{6 \times 3 \sigma }{R \times f \times \rho \times \pi }} \right)^{1/3} $$

Where σ is the standard deviation of the signal background (here: control group cell lysates); R is the sensitivity of the detector for the element of the analyte (cps/μg); *f* is the mass fraction of analyzed metal element in the NPs; ρ is the density of the NPs.

NP number limits of detection (LOD_numberNP_) were calculated by:

$$ {\text{LOD}}_{\text{numberNP}} = 3 \times \frac{1}{{\delta  _{neb} \beta_{sam}  t_{i} }} $$

Where δ_neb_ is the nebulizer transport efficiency; β_sam_ is the sample flow rate; and t_i_ is the total acquisition time.

#### LA-ICP-MS of single cells

LA-ICP-MS was performed using an NWR 213 laser system (Electro Scientific Industries, Huntingdon, UK) coupled to an Element XR sector field ICP-MS (Thermo Fisher Scientific GmbH, Dreieich, Germany). The system was warmed up before analysis and tuned by ablating line scans with 200 µm spot size, 10 µm/s scan rate, 20 Hz repetition rate and 100 % laser energy from a microscope glass slide while optimizing the parameters for high signal intensities.

Glass slides were fixed in the ablation cell which mechanically moves the samples in xyz-direction under the fixed laser. At first, ablation parameters for dried cells were optimized to ensure complete ablation of the cells and a total coverage of the analyzed area which resulted in a scan speed of 5 µm/s, a spot size of 4 µm, a repetition rate of 10 Hz, a laser fluence of about 2.5 mj/cm^2^ and a lane distance of 5 µm, respectively. Details on the used equipment and the optimized parameters are given in Additional file 1: Table S2. To analyze sufficient numbers of cells, three different areas for each treatment and control group were evaluated. The aerosol was transported by a He carrier gas flow from the ablation cell to the ICP-MS where an additional Ar gas flow was introduced prior to the atomization and ionization in the plasma.

For quantification, two dilution series made from TiO_2_ NPs and Ag NPs in an untreated cell lysate matrix were prepared. The cell concentration was adjusted to theoretically 2 cells per µl (Additional file 1: Table S3). These solutions were then digested by adding nitric acid and hydrochloric acid (1:1) in an ultrasonic bath over night at 60 °C. 0.5 µl of each point of these dilution series were pipetted in duplicate onto a glass slide, dried and analyzed by LA-ICP-MS. For the quantification of this calibration series, ablation under the aforementioned parameters would be too time-consuming. Therefore, laser ablation for the calibration series was done with a higher scan speed of 200 µm/s, a bigger spot size of 250 µm and corresponding lane distance of 240 µm, and a higher laser fluence of about 2.5 mJ/cm^2^ (Additional file 1: Table S4).

In parallel, adjacent lines were ablated and the resulting time-dependent ICP-MS scans merged to one data set using lane distance, scan speed and time to calculate the dimensions. Integration of the signal intensity in the pipetted drops and in the cells was done using ImageJ software. Only clearly separated cells were used for integration and analysis. For the calculation of the number of NPs per cell a perfect sphere was assumed for the particles and the DLS size was used. Background correction for the cells and pipetted calibration was done for each slide using areas not containing any cells, cell compartments or droplets.

#### Monitored isotopes

^107^Ag and/or ^109^Ag were detected for the Ag NPs. Despite its low abundance of 5.5 %, ^49^Ti was selected to conduct ICP-MS because it shows a low background noise and polyatomic interferences such as ^33^S^16^O or ^31^P^18^O can be resolved by the collision cell technique (CCT) of quadrupole ICP-MS [20]. Higher abundant and therefore more sensitive ^48^Ti (73.8 %) was selected to conduct SP-ICP-MS [42] and LA-ICP-MS. For SP-ICP-MS, even though polyatomic interferences such as ^32^S^16^O and ^36^Ar^12^C or the isobaric interference with ^48^Ca are possible, these interferences only contribute to the continuous background while the TiO_2_ particles have distinct discontinuous signals and can thus be distinguished and isolated from the background. Our LOD_size_ for ^48^Ti particles was 57 nm (equivalent to 69 nm TiO_2_), which was derived from background noise of cell lysates. Our LOD_size_ was lower than for ^49^Ti (75 nm) and ^47^Ti (93 nm) particles, which was derived from water background by using the same instrument [41]. For LA-ICP-MS, using a sector field ICP-MS allows resolving the polyatomic interferences for ^48^Ti but still does not suffice to distinguish it from ^48^Ca.

#### Statistics

Two-tailed Student’s unpaired t–test was used for two-group comparison. p < 0.05 was considered statistically significant.

## Additional file

10.1186/s12951-016-0203-z Cellular uptake of TiO_2_ and Ag nanoparticles: comparison of different ICP-MS techniques.

## Abbreviations

AAS: atomic absorption spectrometry
CCM: cell culture medium
CCT: collision cell technique
DLS: dynamic light scattering
DPBS: Dulbecco’s phosphate buffered saline
EDX: energy-dispersive X-ray spectroscopy
HUVECs: human umbilical vein endothelial cells
IBMX: 3-isobutyl-1-methylxanthine
ICP-MS: inductively coupled plasma mass spectrometry
ICP-OES: inductively coupled plasma optical emission spectrometry
LA-ICP-MS: laser ablation ICP-MS
LOD: limit of detection
NPs: nanoparticles
SC-ICP-MS: single cell ICP-MS
SP-ICP-MS: single particle ICP-MS
SEM: scanning electron microscopy
TEM: transmission electron microscopy
