# Supplementary material for: Quantification and visualization of cellular uptake of TiO2 and Ag nanoparticles: comparison of different ICP-MS techniques
Source: J Nanobiotechnology. 2016 Jun 22;14:50. doi: 10.1186/s12951-016-0203-z (PMC4918130; doi:10.1186/s12951-016-0203-z)
Supplement: Supplementary file 1 — 10.1186/s12951-016-0203-z Cellular uptake of TiO2 and Ag nanoparticles: comparison of different ICP-MS techniques. [file 12951_2016_203_MOESM1_ESM.docx]

**Supplementary information**

Quantification and visualization of cellular uptake of TiO_2_ and Ag nanoparticles: Comparison of different ICP-MS techniques

I-Lun Hsiao^1,2^, Frank S. Bierkandt^3^, Philipp Reichardt^1^, Andreas Luch^1^, Yuh-Jeen Huang^2^, Norbert Jakubowski^3^, Jutta Tentschert^1*^, Andrea Haase^1*^

^1^German Federal Institute for Risk Assessment (BfR), Department of Chemical and Product Safety, Berlin, Germany

^2^National Tsing Hua University, Department of Biomedical Engineering and Environmental Sciences, Taiwan

^3^German Federal Institute for Materials Research and Testing (BAM), Division of Inorganic Trace Analysis, Berlin, Germany

*both authors contributed equally

Corresponding author:

Dr. Andrea Haase

German Federal Institute for Risk Assessment (BfR)

Department of Chemical and Product Safety

Max-Dohrn-Strasse 8-10,

10589 Berlin

[andrea.haase@bfr.bund.de](mailto:andrea.haase@bfr.bund.de)


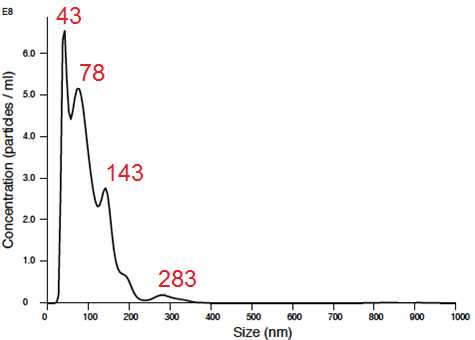

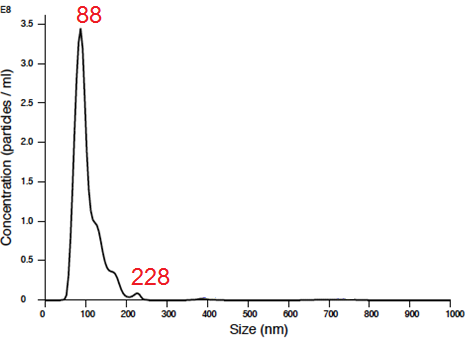


a

b

c

d


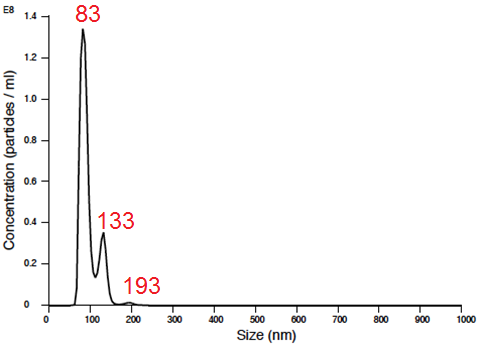

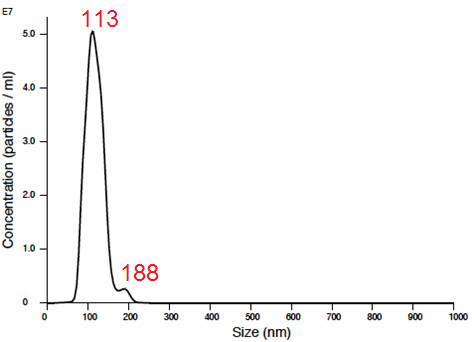


Figure S1. Size distribution of TiO_2_ 7 nm (a); TiO_2_ 20 nm (b); Ag 50 nm (c); Ag 75 nm (d) in complete cell culture medium (CCM) as measured by NTA. Unit of Y-axis was 10^8^ particles/mL for (a) to (c) and 10^7^ particles/mL for (d).

Figure S2. Assessment of cytotoxicity of Ag and TiO_2_NPs in Neuro-2a cells after 24 h of exposure using WST-1 assay.


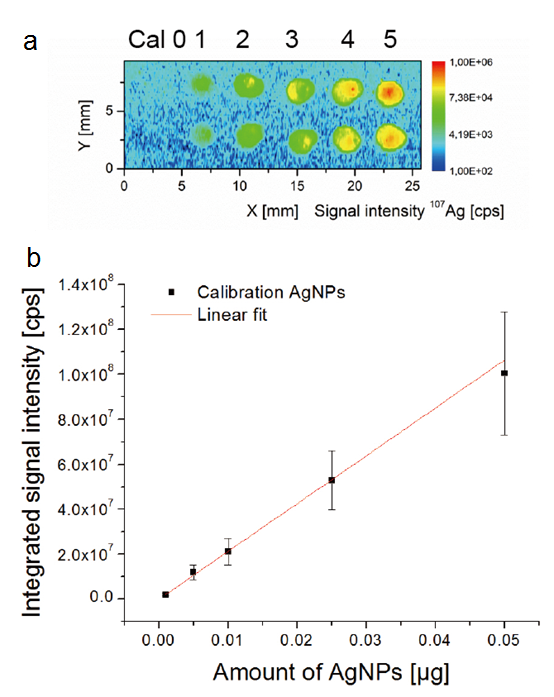


Figure S3. (a) Contour plot for ^107^Ag of the pipetted serial dilution after digestion in duplicate showing from left to right drops with the following amounts of Ag NP: control, 0.001, 0.005, 0.01 0.025 and 0.05 µg. (b) Correlation between the integrated signal intensity and the amount of silver in the drops of the calibration plot (R^2^ = 0.995).


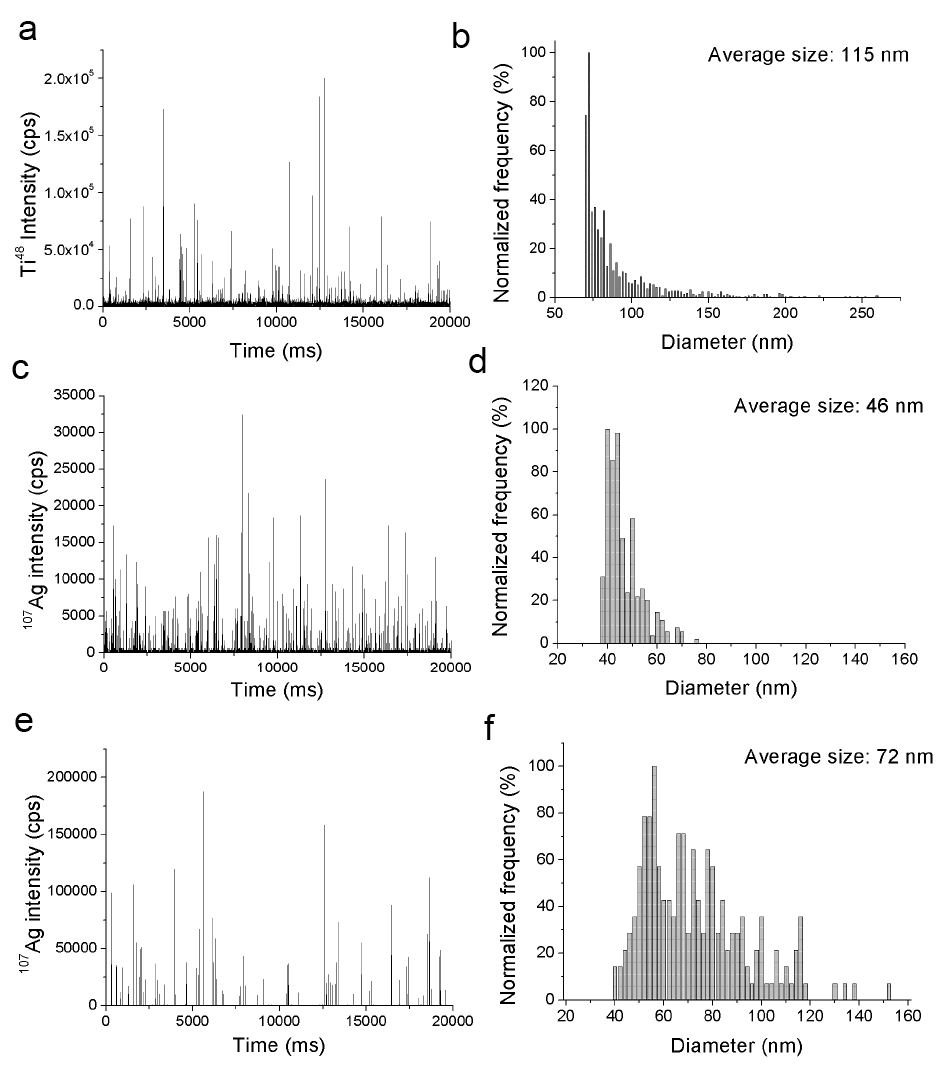


Figure S4. SP-ICP-MS measurement of NPs in water. Raw signal SP-ICP-MS spectra of (a) TiO_2_ 20 nm; (c) Ag 50 nm and (e) Ag 75 nm within 20 000 ms and the corresponding size distribution histograms (b, d, f). Bin width: 2 nm.


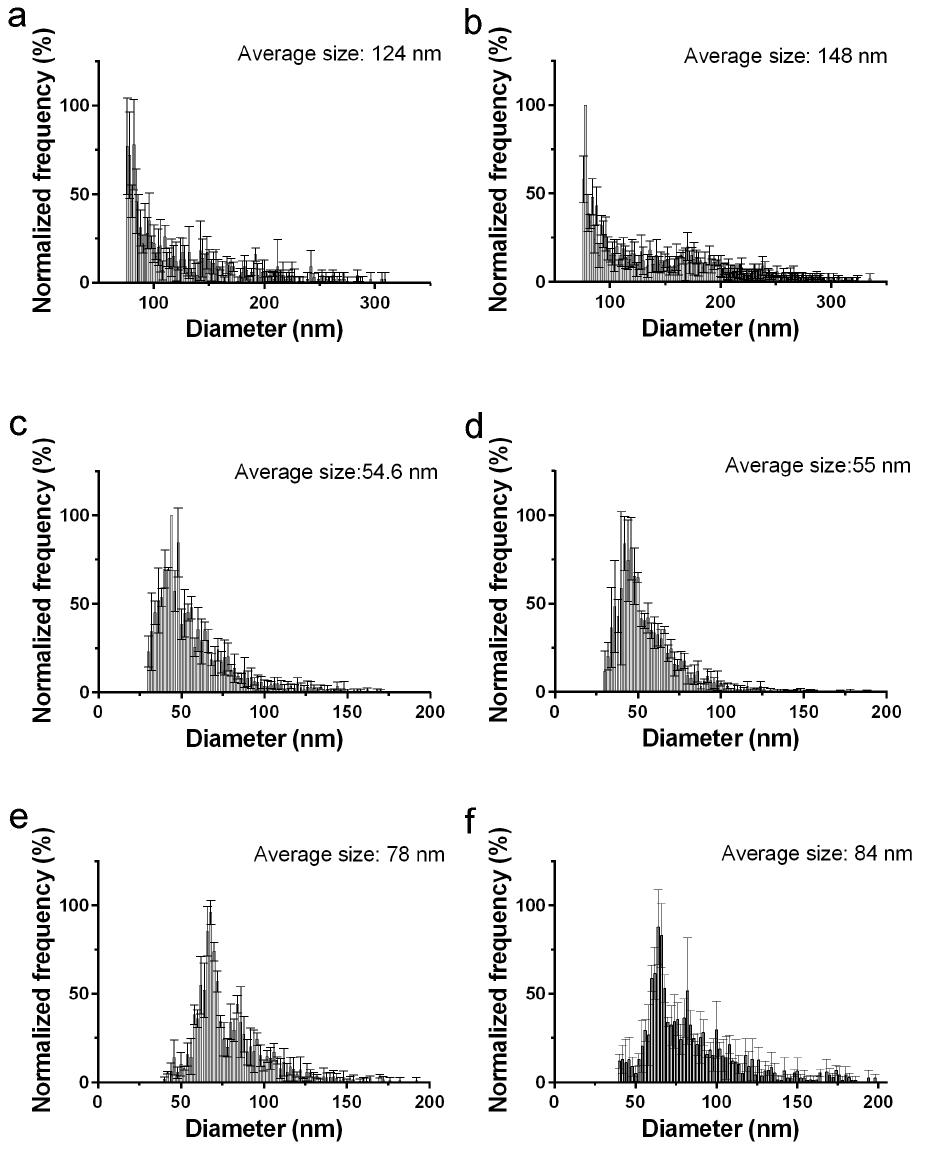


Figure S5. Average size and size distribution of NPs in Neuro-2a cells as analyzed by SP-ICP-MS. (a) TiO_2_ 20 nm, 2 μg/mL; (b) TiO_2_ 20 nm, 10 μg/mL; (c) Ag 50 nm, 2 μg/mL; (d) Ag 50 nm, 10 μg/mL; (e) Ag 75 nm, 2 μg/mL; and (f) Ag 75 nm, 10 μg/mL. Bin width: 2 nm

| **ICP-MS parameters** | |
| --- | --- |
| **Instrument** | **XSeries 2 (Thermo Fisher)** |
| **RF power** | **1400 W** |
| **Cones** | **Ni skimmer and sampler** |
| **Additional gas flow (Ar)** | **0.95 L min^-1^** |
| **Isotopes** | **^49^Ti, ^107^Ag, ^115^In (Internal standard)** |
| **Operation mode** | **Continuous** |
|  |  |
| **SP-ICP-MS parameters** | |
| **Instrument** | **XSeries 2 (Thermo Fisher)** |
| **RF power** | **1400 W** |
| **Cones** | **Ni skimmer and sampler** |
| **Additional gas flow (Ar)** | **0.95 L min^-1^** |
| **Isotopes** | **^107^Ag , ^48^Ti, ^197^Au (AuNP standard)** |
| **Operation mode** | **Spike** |
| **Sample flow rate** | **0.34 mL/min** |
| **Dwell time** | **3 ms** |
| **Duration time** | **1 min** |

Table S1. Instruments and parameters for analyzing NPs in digested cells

and cell lysates.


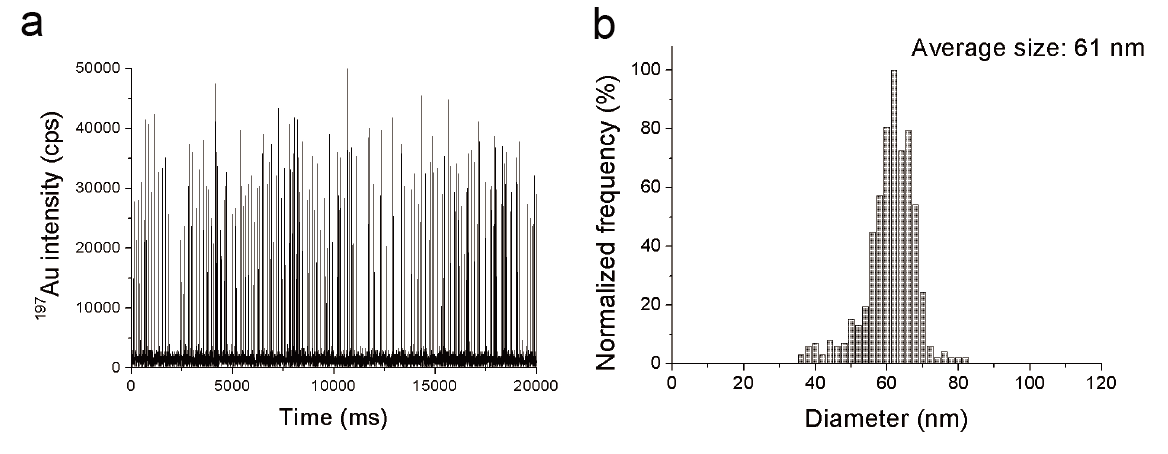


Figure S6. SP-ICP-MS measurement of the 60 nm AuNP standard (a) raw signal spectra of SP-ICP-MS within 20 000 ms, and (b) the corresponding size distribution histograms. Data were measured at 100 ppt. Bin width: 2 nm

| **ICP-MS parameters** | |
| --- | --- |
| **Instrument** | **Element XR (Thermo Fisher)** |
| **RF power** | **1350 W** |
| **Cones** | **Ni skimmer and sampler** |
| **Additional gas flow (Ar)** | **0.5 – 0.65 L min^-1^** |
| **Resolution** | **LR** |
| **Isotopes** | **^48^Ti, ^107^Ag, ^109^Ag,** |
|  |  |
| **LA parameters** | |
| **Instrument** | **ESI NWR213 (Nd:YAG Laser)** |
| **Scan rate** | **5 µm s^-1^** |
| **Spot size** | **4 µm** |
| **Repetition rate** | **10 Hz** |
| **Lane distance** | **5 µm** |
| **Carrier gas (He)** | **1. L min^-1^** |
| **Laser energy** | **~50% (2.5 mJ/cm^-1^)** |
| **Pulse width** | **< 4 ns** |

Table S2. Instruments and parameters for laser ablation of dried cells.

| **Sample** | **Cells per drop** | **Amount of Ag per drop** |
| --- | --- | --- |
|  |  | [µg] |
| **Cal 0** | 1 | 0 |
| **Cal 1** | 1 | 0.001 |
| **Cal 2** | 1 | 0.005 |
| **Cal 3** | 1 | 0.010 |
| **Cal 4** | 1 | 0.025 |
| **Cal 5** | 1 | 0.050 |

Table S3. Final amount of silver and cells per 0.5 µl drop for the serial dilution.

| **ICP-MS parameters** | |
| --- | --- |
| **Instrument** | **Element XR (Thermo Fisher)** |
| **RF power** | **1350 W** |
| **Cones** | **Ni skimmer and sampler** |
| **Additional gas flow (Ar)** | **0.5 – 0.65 L min^-1^** |
| **Resolution** | **LR** |
| **Isotopes** | **^48^Ti, ^107^Ag, ^109^Ag,** |
|  |  |
| **LA parameters** | |
| **Instrument** | **ESI NWR213 (Nd:YAG Laser)** |
| **Scan rate** | **200 µm s^-1^** |
| **Spot size** | **250 µm** |
| **Repetition rate** | **20 Hz** |
| **Lane distance** | **240 µm** |
| **Carrier gas (He)** | **1.0 L min^-1^** |
| **Laser energy** | **~50% (2.5 mJ/cm^-1^)** |
| **Pulse width** | **< 4 ns** |

Table S4. Instruments and parameters for laser ablation of dried

drops of the serial dilution.
